# From Common Pathway to Divergent Diseases: Metabolic Aspects of Inborn Errors of CoA Biosynthesis

**DOI:** 10.1002/jimd.70239

**Published:** 2026-08-15

**Authors:** Ivano Di Meo, Yair Anikster, Valeria Tiranti, Arcangela Iuso

**Affiliations:** ^1^ Unit of Medical Genetics and Neurogenetics Fondazione IRCCS Istituto Neurologico Carlo Besta Milan Italy; ^2^ Gray Faculty of Medical and Health Sciences Tel Aviv University Tel Aviv Israel; ^3^ Metabolic Disease Unit Edmond and Lily Safra Children's Hospital, Sheba Medical Center Ramat Gan Israel; ^4^ Institute of Neurogenomics, Helmholtz Zentrum München Neuherberg Germany; ^5^ Institute of Human Genetics, School of Medicine and Health Technical University of Munich Munich Germany

**Keywords:** COASY, coenzyme A (CoA), neurodegeneration with brain iron accumulation (NBIA), PANK2, PPCDC, PPCS

## Abstract

Coenzyme A (CoA) biosynthesis is a conserved, dynamically regulated pathway essential for mitochondrial energy production, fatty acid oxidation, lipid biosynthesis and protein acylation. Biallelic variants in *PANK2*, *PPCS*, *PPCDC*, and *COASY* cause rare inborn errors of CoA biosynthesis, associated with markedly different clinical phenotypes: *PANK2* and *COASY* defects predominantly cause neurological disorders within or adjacent to the neurodegeneration with brain iron accumulation (NBIA) spectrum, whereas *PPCS* and *PPCDC* deficiencies present mainly as severe early‐onset dilated cardiomyopathy. However, *COASY* variants can also cause pontocerebellar hypoplasia and riboflavin‐responsive lipid storage myopathy. This review examines these four disorders from a metabolic perspective, integrating clinical features, experimental models, biochemical data and emerging therapeutic approaches. Current evidence indicates that disease pathogenesis cannot be explained only by global CoA depletion. Total CoA levels may be reduced in *PPCS* and *PPCDC* deficiency, but are often preserved under basal conditions in PKAN and COASY‐related models. Instead, impaired compartment‐specific CoA handling and failure to sustain CoA‐dependent flux under increased metabolic demand are emerging as central pathogenic concepts. Perturbation of fatty acid handling, acyl‐CoA/acylcarnitine balance, mitochondrial function, iron homeostasis, protein acylation and 4′‐phosphopantetheinylation may contribute to tissue‐selective vulnerability. Therapeutic strategies are therefore likely to require disease‐specific approaches, including precursor bypass or PANK activation where pathway flux can be restored, early pantethine supplementation in cardiomyopathic forms, and downstream or gene‐directed strategies for COASY‐related disorders. Understanding CoA as a regulator of metabolic adaptability provides a unifying framework for interpreting both shared mechanisms and disease divergence.

## 1 Introduction

**FIGURE 1:**
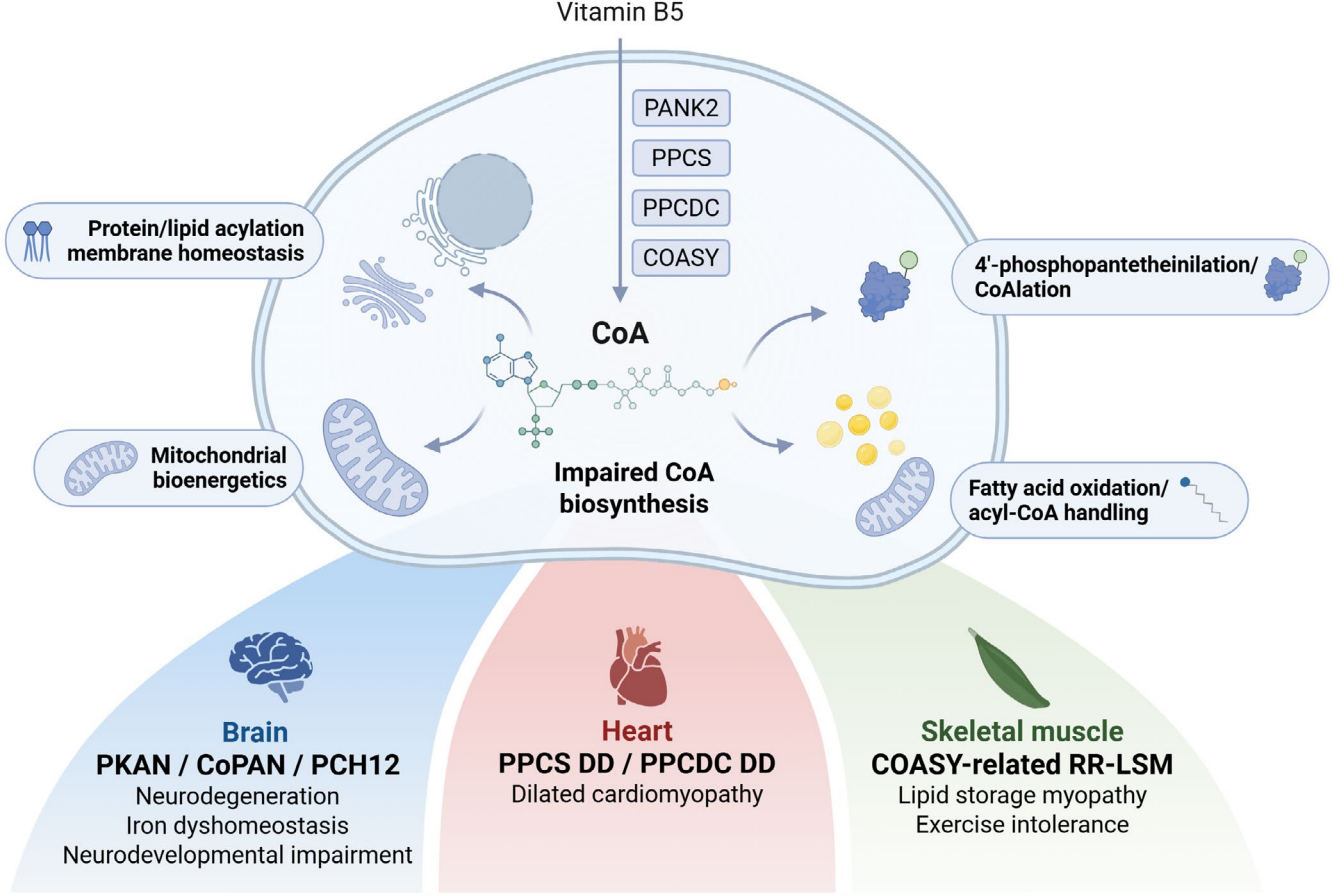
Metabolic consequences and tissue‐specific manifestations of impaired CoA biosynthesis. Coenzyme A is synthesized from pantothenate (vitamin B5) through a conserved pathway involving PANK2, PPCS, PPCDC, and COASY. Pathogenic variants in these enzymes impair CoA biosynthesis and affect CoA‐dependent processes, including mitochondrial bioenergetics, fatty acid oxidation and acyl‐CoA handling, protein and lipid acylation, membrane homeostasis, 4′‐phosphopantetheinylation and protein CoAlation. Despite affecting the same pathway, these disorders show distinct tissue vulnerability: *PANK2* and *COASY* variants mainly affect the nervous system, causing PKAN, CoPAN, and PCH12; *PPCS* and *PPCDC* deficiencies primarily cause early‐onset dilated cardiomyopathy; selected *COASY* variants are associated with RR‐LMS. The figure highlights impaired CoA‐dependent metabolic adaptability as a unifying concept across these divergent phenotypes. CoA, coenzyme A; CoPAN, COASY protein‐associated neurodegeneration; DD, deficiency disorder; PCH12, pontocerebellar hypoplasia type 12; PKAN, pantothenate kinase‐associated neurodegeneration; RR‐LSM, riboflavin‐responsive lipid storage myopathy.

Coenzyme A (CoA) is an essential cofactor required for a broad range of metabolic processes, including mitochondrial energy production, fatty acid oxidation (FAO), lipid biosynthesis, and protein posttranscriptional modification (Figure 1) [1]. Beyond its classical biochemical role, CoA has emerged as a key integrator of cellular metabolism, linking nutrient availability to multiple metabolic and regulatory pathways [2]. Given its central function, disruption of CoA homeostasis is predicted to affect multiple cellular processes and systems.

**FIGURE 2:**
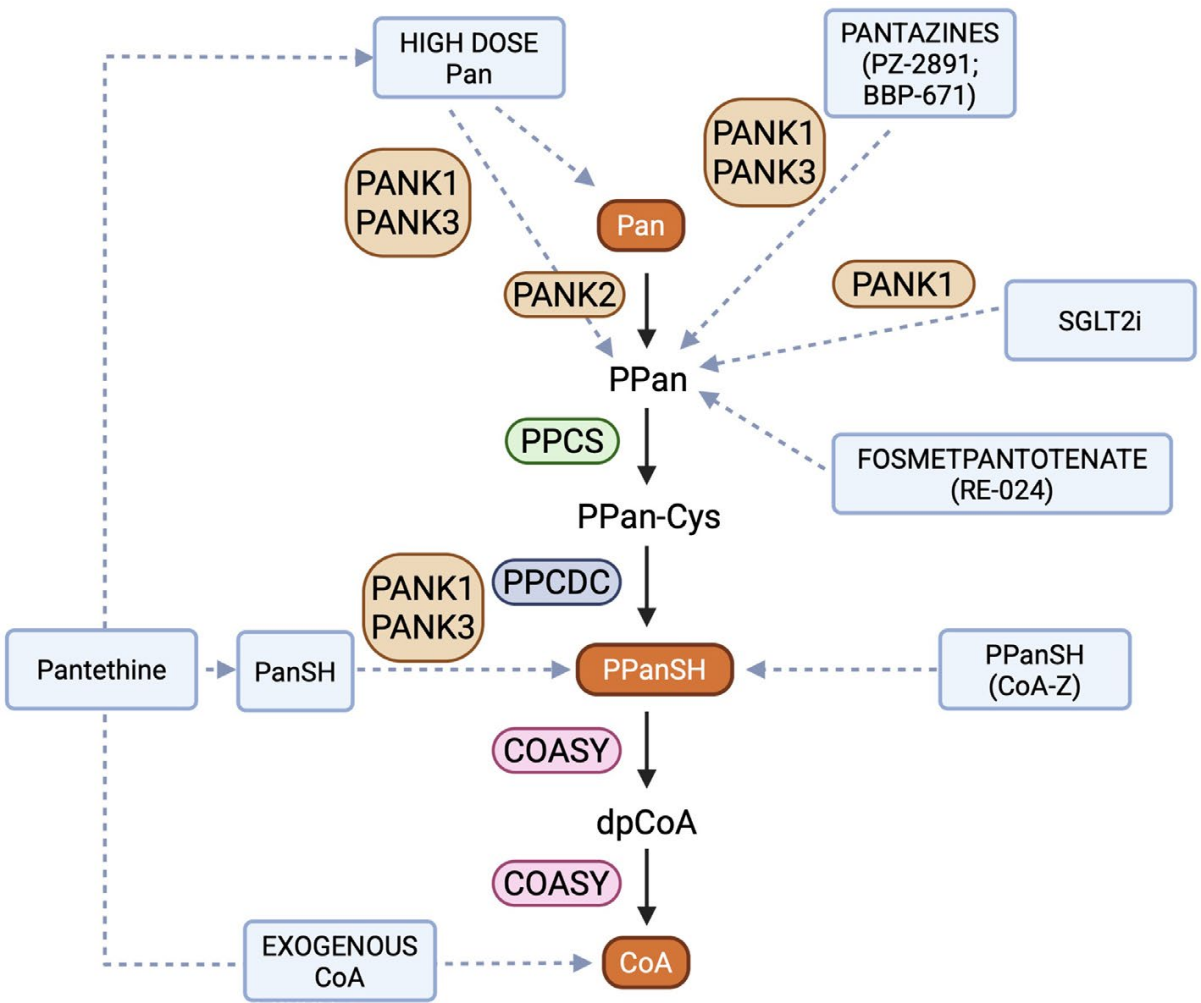
Pathway‐directed strategies to restore CoA biosynthesis. The de novo pathway converts pantothenate into CoA through five sequential reactions catalyzed by PANK1–3, PPCS, PPCDC, and the bifunctional enzyme COASY. Proposed therapeutic approaches include supplementation with pathway precursors or intermediates, such as high‐dose pantothenate, fosmetpantotenate, pantethine, and PPanSH; pharmacological activation of compensatory PANK isoforms by pantazines or SGLT2 inhibitors; and exogenous CoA administration. Black arrows indicate the reactions of de novo CoA biosynthesis, whereas dashed arrows indicate the proposed metabolic entry points or enzymatic targets of each intervention. CoA, coenzyme A; COASY, CoA synthase; dpCoA, dephospho‐CoA; Pan, pantothenate; PANK, pantothenate kinase; PanSH, pantetheine; PPan, 4′‐phosphopantothenate; PPan‐Cys, 4′‐phospho‐N‐pantothenoylcysteine; PPanSH, 4′‐phosphopantetheine; PPCDC, phosphopantothenoylcysteine decarboxylase; PPCS, phosphopantothenoylcysteine synthetase; SGLT2i, sodium–glucose cotransporter 2 inhibitors.

De novo CoA biosynthesis proceeds through five sequential enzymatic reactions (Figure 2) that convert pantothenate (vitamin B5), cysteine, and ATP into CoA, with each step introducing a specific chemical feature required for its function as an acyl carrier [3, 4]. The pathway is initiated by pantothenate kinase (PANK), which catalyzes the ATP‐dependent phosphorylation of pantothenate to 4′‐phosphopantothenate and constitutes the main rate‐limiting step, tightly regulated by feedback inhibition from CoA and its thioesters. Phosphopantothenoylcysteine synthetase (PPCS) then condenses this intermediate with cysteine, followed by decarboxylation by phosphopantothenoylcysteine decarboxylase (PPCDC) to generate 4′‐phosphopantetheine. The final steps are catalyzed by the bifunctional enzyme CoA synthase (COASY), which converts 4′‐phosphopantetheine into dephospho‐CoA and subsequently into CoA. Although often depicted as a linear pathway, CoA biosynthesis is dynamically regulated through feedback mechanisms, subcellular compartmentalization, and precursor availability, which collectively determine pathway flux across different cellular contexts [5].

Biallelic pathogenic variants in the respective genes, namely *PANK2*, *PPCS*, *PPCDC*, and *COASY*, cause distinct monogenic disorders, which are collectively classified as inborn errors of CoA biosynthesis [6]. Despite affecting a linear metabolic pathway, these disorders present with striking clinical heterogeneity (Table 1). Pathogenic variants in *PANK2* and *COASY* are associated with neurodegeneration with brain iron accumulation (NBIA), characterized by progressive movement disorders and iron deposition in the basal ganglia [7]. In contrast, variants in *PPCS* and *PPCDC* predominantly result in severe early‐onset dilated cardiomyopathy, often with rapid progression and high mortality in the absence of overt neurodegeneration [8, 9]. More recently, *COASY* variants have also been associated with riboflavin‐responsive metabolic myopathy, further expanding the phenotypic landscape beyond the central nervous system [10]. This clinical divergence suggests that the consequences of impaired CoA biosynthesis are strongly context‐dependent and shaped by tissue‐specific metabolic demands, challenging the prevailing view that a uniform reduction in CoA levels is sufficient to explain disease pathogenesis.

**TABLE 1:** Clinical, metabolic, and therapeutic features of inborn errors of CoA biosynthesis.

Gene/enzyme	Pathway step	Main phenotype	Involved tissues	Total CoA readout	Emerging pathogenic concepts	Therapeutic implications
PANK2/pantothenate kinase 2	First, rate‐limiting step; pantothenate to 4′‐phosphopantothenate	PKAN (NBIA), typical, or atypical phenotype	CNS, particularly basal ganglia; retina in selected cases	Often preserved under basal conditions	Mitochondrial lipid metabolism, mtACP phosphopantetheinylation, iron dyshomeostasis, and oxidative stress are implicated; impaired CoA‐dependent flux during lipid or FAO stress may contribute	4′‐Phosphopantetheine and PANK activators are pathway‐directed approaches; downstream strategies targeting iron handling, mitochondrial dysfunction, lipid metabolism, or PPARγ‐related pathways may be useful, but clinical efficacy remains uncertain
PPCS/phosphopantothenoylcysteine synthetase	Second step; 4′‐phosphopantothenate plus cysteine to phosphopantothenoylcysteine	Early‐onset dilated cardiomyopathy; systemic metabolic decompensation in severe cases	Heart, possible neuromuscular involvement	Reduced in patient‐derived fibroblasts and cardiac models	Direct CoA biosynthetic defect; insufficient CoA supply during cardiomyocyte maturation and high oxidative demand	Strong rationale for pantethine, with early treatment likely critical; 4′‐phosphopantetheine remains a mechanistically plausible candidate
PPCDC/phosphopantothenoylcysteine decarboxylase	Third step; phosphopantothenoylcysteine to 4′‐phosphopantetheine	Severe neonatal or early infantile dilated cardiomyopathy; lactic acidosis, hyperammonemia and liver involvement may occur	Heart	Reduced in patient‐derived fibroblasts	Reduced CoA availability with secondary mitochondrial dysfunction and impaired fatty acid handling	Pantethine and 4′‐phosphopantetheine are mechanistically rational bypass strategies, although evidence remains anecdotal
COASY/CoA synthase	Final two steps; 4′‐phosphopantetheine to dephospho‐CoA and then CoA	CoPAN (NBIA), typical or atypical phenotype; pontocerebellar hypoplasia; riboflavin‐responsive lipid storage myopathy	CNS, particularly basal ganglia; developing brain; skeletal muscle	Often preserved in some patient‐derived models, but reduced in selected experimental systems	Impaired final CoA synthesis; compartment‐specific CoA handling; reduced mitochondrial 4′‐phosphopantetheinylation; mitochondrial dysfunction; lipid and iron dyshomeostasis	No established disease‐modifying therapy for CoPAN or PCH12; precursor bypass depends on residual COASY activity; riboflavin may benefit selected hypomorphic variants; downstream PPARγ activation, gene‐based strategies and stabilization of hypomorphic variants remain possible directions

A central unresolved issue in the field is the relationship between CoA metabolism and disease pathogenesis. Several experimental models and patient‐derived cells do not consistently show a marked reduction in total CoA levels [11, 12, 13, 14, 15], raising questions about whether global CoA deficiency alone is sufficient to explain disease mechanisms. Instead, emerging evidence points toward more complex scenarios involving compartment‐specific alterations, context‐dependent metabolic stress, and selective vulnerability of specific cell types. CoA is not distributed as a homogeneous cellular pool, but is partitioned across mitochondria, cytosol, peroxisomes, and nucleus, with each compartment supporting distinct biochemical processes [1, 16]. Moreover, the demand for CoA changes considerably with nutrient availability, developmental stage, and cellular state, suggesting that the ability to sustain CoA‐dependent metabolic flux, rather than steady‐state CoA abundance, may represent the functionally limiting factor [17]. A model centered solely on bulk CoA depletion therefore fails to account for the selective vulnerability of specific tissues and pathways. Notably, the differential impact of pathway position is itself informative: mid‐pathway defects in PPCS and PPCDC preferentially affect the heart, whereas defects in the initial (PANK2) and final (COASY) steps predominantly affect the nervous system, suggesting that tissue‐specific metabolic requirements outweigh pathway topology in determining disease manifestation.

An alternative model, increasingly supported by recent evidence, holds that inherited defects in CoA biosynthesis impair the capacity of cells to sustain CoA‐dependent metabolic processes under conditions of increased demand. Within this model, disease arises not from a static deficit of CoA, but from a failure to maintain adequate flux through critical reactions, including mitochondrial FAO, protein acylation, and 4′‐phosphopantetheinylation of metabolic enzymes, when metabolic needs increase [5, 18]. In addition, CoA can participate in redox‐sensitive protein CoAlation, a reversible cysteine modification triggered by oxidative or metabolic stress; however, its role in inherited disorders of CoA biosynthesis remains to be elucidated [19, 20].

Taken together, these observations argue against a unifying model based solely on reduced CoA synthesis and instead support a conceptual framework in which CoA functions as a regulator of metabolic adaptability. In this view, inherited defects in CoA biosynthesis compromise the ability of cells to respond to fluctuating metabolic demands, leading to selective failure of critical biochemical processes in vulnerable tissues. The key challenge is therefore not simply to quantify CoA levels, but to identify which CoA‐dependent reactions become limiting, under which conditions, and in which cellular contexts.

In this review, we examine the four genetic disorders of CoA biosynthesis through this lens, analyzing for each gene the relationship between clinical phenotype, metabolic perturbation, and pathogenic mechanism. Rather than treating these conditions as variations on a single biochemical theme, we emphasize both their shared features and their divergence, with the aim of defining a coherent mechanistic model to guide future studies and therapeutic strategies.

## 2 PANK2

### 2.1 Clinical Aspects

Pathogenic variants in *PANK2* cause pantothenate kinase‐associated neurodegeneration (PKAN; OMIM #234200), one of the most common forms of NBIA [21]. PKAN is inherited as an autosomal recessive disorder and has an estimated prevalence of approximately 1–2 cases per million [22, 23]. Clinically, it is traditionally divided into classic and atypical forms. Classic PKAN usually manifests in the first decade of life with a rapid progressive course, whereas atypical PKAN has later onset and slower progression. Both forms are characterized by progressive dystonia, spasticity, parkinsonism, gait impairment, dysarthria, cognitive involvement, pigmentary retinopathy, and neuropsychiatric manifestations [24]. Brain MRI classically shows the “eye‐of‐the‐tiger” sign, consisting of bilateral pallidal hypointensity with a central hyperintense signal on T2‐weighted images, reflecting iron accumulation, calcification, and tissue damage in the *globus pallidus* (GP) [25, 26]. Although highly suggestive, this sign should not be considered absolutely pathognomonic, as it may be absent in genetically confirmed PKAN and has been reported in other NBIA disorders [27, 28]. Postmortem studies show predominant involvement of the GP, with neuronal loss, vacuolization, axonal spheroids, neuroaxonal dystrophy, ubiquitinated protein aggregates, reactive astrogliosis, microglial activation, and iron accumulation in neurons and astrocytes [29, 30].

### 2.2 Metabolic Aspects

PANK2 catalyzes the first, rate‐limiting step of de novo CoA biosynthesis, converting pantothenate into 4′‐phosphopantothenate. Among human PANK isoforms, PANK2 is the only associated with mitochondria, particularly in the intermembrane space, and its regulation appears adapted to mitochondrial lipid metabolism [31]. PANK2 is highly sensitive to feedback inhibition by acetyl‐CoA and long‐chain acyl‐CoAs, although this can be relieved by palmitoyl‐carnitine [32]. The mutational spectrum of *PANK2* is broad, and residual enzymatic function may contribute to disease severity, although genotype–phenotype correlations remain inconclusive [33, 34].

A major unresolved issue in PKAN pathogenesis is that total cellular CoA levels are not consistently reduced in patient fibroblasts, PANK2‐deficient cell lines, or *Pank2* knockout mouse tissues under basal, glucose‐replete conditions [11, 12, 35]. This has challenged the simple model in which PKAN results from constitutive global CoA depletion. Recent preprint data by Nordlie et al. offer a potential alternative interpretation. Using labeled pantothenate tracing, the authors showed that PANK2, but not PANK1 or PANK3, is required to maintain de novo CoA synthesis during the switch to FAO. Under palmitate stress, PANK2‐deficient cells showed impaired CoA and acetyl‐CoA production, energetic failure, fatty acid accumulation, autophagy activation, and reduced viability [36]. Consistent with this emerging model, recent observations have documented systemic metabolic abnormalities in PKAN patients. A small Turkish cohort reported a CPT1‐like acylcarnitine profile in patients with classic PKAN, characterized by elevated free carnitine (C0) and/or increased C0/(C16 + C18) ratio, providing a potential early biochemical clue and linking PKAN to impaired fatty acid handling [37]. Mechanistically, this profile may reflect impaired fatty acid activation, with accumulation of free carnitine and relative reduction of long‐chain acylcarnitines, thereby metabolically mimicking CPT1 deficiency. Moreover, plasma and serum metabolomic studies reported increased markers of mitochondrial dysfunction, reduced lipid species, oxidative stress, and ferroptosis in PKAN patients [38, 39], while a single case report also described dyslipidemia and hypercalciuria in a patient with a novel *PANK2* variant [40]. Together, these findings suggest that the metabolic consequences of PANK2 deficiency extend beyond the CNS and may become detectable when CoA‐dependent lipid metabolism is challenged. If confirmed, this may explain why PANK2 deficiency may remain biochemically silent under basal conditions but become pathogenic during states of increased lipid‐dependent energetic demand.

Several downstream mechanisms may connect impaired PANK2 function to neurodegeneration. Altered CoA availability can impair phosphopantetheinylation of mitochondrial acyl carrier protein (mtACP/NDUFAB1), affecting mitochondrial fatty acid synthesis, lipoic acid production, Fe‐S cluster biogenesis, respiratory complex assembly, and pyruvate dehydrogenase activity, all converging on mitochondrial dysfunction, oxidative stress, and defective energy metabolism [18]. This mechanism is particularly relevant because it links CoA biosynthesis to mitochondrial processes that depend on 4′‐phosphopantetheine as a prosthetic group, rather than only on the availability of free CoA. PANK2 has also been linked to mitochondrial quality control through its functional interaction with PINK1, where local acetyl‐CoA metabolism may regulate mitophagy via p62/SQSTM1 acetylation [41]. Although this pathway provides an additional connection between CoA metabolism and neuronal vulnerability, its contribution to human PKAN remains to be fully defined. In parallel, altered CoA‐dependent acylation may contribute to iron dyshomeostasis. Impaired palmitoylation of transferrin receptor 1 (TfR1) disrupts receptor trafficking and recycling, promoting sustained iron uptake [42]. PKAN astrocytes also show abnormal endosomal trafficking and increased transferrin uptake [43]. These mitochondrial and iron‐related defects are supported by disease models, although each model captures only part of the human disorder. *Pank2* knockout mice show mitochondrial abnormalities, dopamine metabolism defects, retinal degeneration, infertility, and iron perturbation, but they do not fully reproduce progressive human neurodegeneration or suffer from any clinical sign [44, 45, 46]. Dietary stressors, including ketogenic or pantothenate‐deficient diets, can unmask neuromuscular phenotypes, again supporting a stress‐dependent model [47]. Human iPSC‐derived neurons and astrocytes appear more informative, showing iron dyshomeostasis, mitochondrial dysfunction, oxidative stress, lipids peroxidation, ferroptosis susceptibility, and marked iron accumulation, particularly in astrocytes and GABAergic neurons [12, 43, 48, 49].

### 2.3 Therapeutic Approaches

Therapeutic strategies have largely aimed to bypass the enzymatic block, increase CoA synthesis, or target downstream pathology (Figure 2). Pantothenate supplementation may benefit cells with residual PANK2 activity, but clinical benefit has not been demonstrated [50]. Pantethine showed partial rescue in Drosophila, zebrafish, and mouse PKAN models under metabolic stress [47, 51, 52, 53, 54], but an open‐label clinical trial in children showed tolerability without significant motor improvement, although a slowing of progression was suggested [55]. Fosmetpantothenate, a phosphopantothenate prodrug, restored CoA‐related defects in cellular models and had favorable pharmacokinetics, but a randomized phase III trial did not demonstrate clinical efficacy [56, 57, 58]. Downstream intermediates appear more promising conceptually. 4′‐phosphopantetheine bypasses the PANK2 block by entering CoA biosynthesis downstream, corrects CoA‐related and downstream defects in preclinical models, and is currently being evaluated in clinical trials (NCT04182763) [46, 59]. Acetyl‐4′‐phosphopantetheine shows improved stability and rescues phenotypes in flies and mice, but remains preclinical [60]. A small pilot study using a cocktail of pantothenate, pantethine, omega‐3 fatty acids, and vitamin E reported improved cellular readouts and clinical stabilization in three patients, but the uncontrolled design and combined intervention make efficacy difficult to interpret [61].

A second strategy targets PANK regulation directly. Allosteric PANK activators, including pantazines and BBP‐671, aim to relieve acyl‐CoA‐mediated feedback inhibition and increase CoA synthesis through residual or alternative PANK activity. However, although PANK activators provide strong preclinical proof‐of‐concept that CoA biosynthesis can be pharmacologically enhanced, their efficacy in PKAN patients remains unproven [62, 63, 64, 65, 66]. Conceptually related, SGLT2 inhibitors directly activate PANK1 in human cardiac tissue and stimulate CoA synthesis [67]. While not a PKAN therapy, this finding demonstrates that pharmacological PANK activation is feasible in human tissues.

Other approaches target downstream consequences rather than the primary defect. Deferiprone reduces brain iron on MRI but has limited and inconsistent clinical impact, indicating that iron accumulation is an important disease feature but unlikely to be the sole driver of progression [68, 69, 70]. Leriglitazone, a brain‐penetrant PPARγ agonist, improved viability, mitochondrial respiration, and iron handling in PKAN iPSC‐derived astrocytes [71]. These data suggest that supplementing precursors alone is unlikely to be effective for most patients. Instead, approaches that restore pathway flux downstream of PANK2, activate compensatory PANK enzyme, or target shared downstream mechanisms, alone or in combination, may offer a more promising disease‐modifying strategy.

## 3 PPCS

### 3.1 Clinical Aspects

Pathogenic variants in *PPCS* cause phosphopantothenoylcysteine synthetase deficiency (PPCS deficiency; OMIM #618189), also referred to as CMD2C, an ultra‐rare autosomal recessive disorder of CoA biosynthesis [8]. Only 13 patients have been reported to date, with early‐onset dilated cardiomyopathy (DCM) as the core and most consistent clinical feature [8, 9, 72, 73, 74]. DCM typically presents in the first months to early years of life and can be rapidly fatal without intervention, often requiring heart transplantation or metabolic rescue.

Extracardiac manifestations are variably present and include hypotonia, rhabdomyolysis, dysmorphic features, and neuromuscular involvement, with some patients developing neurological deterioration [73]. Severe infantile presentations may also include systemic metabolic decompensation, including ketoacidosis, hyperammonemia, lactic acidosis, rhabdomyolysis, feeding difficulties, and gastroparesis, supporting classification of PPCS deficiency as a systemic inborn error of metabolism rather than an isolated cardiomyopathy [74]. Unlike PANK2‐ or COASY‐related disorders, PPCS deficiency is not associated with brain iron accumulation on MRI. Where observed, brain volume loss appears secondary to cardiocirculatory compromise, ECMO, or resuscitation rather than primary neurodegeneration [73].

Exploratory genotype–phenotype correlations suggest that variants affecting exons 1–2 associate with DCM plus neuromuscular features, while p.Glu233Val in exon 3 has been linked to isolated DCM with variable expressivity [73]. Biallelic complete loss‐of‐function appears incompatible with life, as all reported patients retain residual PPCS activity. Heart transplantation has succeeded without recurrence in at least one case, implying predominant cardiac isoform relevance [73].

### 3.2 Metabolic Aspects

PPCS catalyzes the second step of de novo CoA biosynthesis, forming phosphopantothenoylcysteine from 4′‐phosphopantothenate and cysteine. The cytosolic protein functions as a homodimer, and critical oligomerization domains appear important for protein stability and function [1, 75]. All identified pathogenic variants destabilize PPCS protein, as evidenced by reduced protein levels in fibroblasts, iPSCs, cardiac progenitors, and cardiomyocytes, without corresponding reduction in mRNA levels, indicating posttranslational instability as the primary pathogenic mechanism [73].

A critical metabolic distinction sets PPCS deficiency apart from PANK2‐ and COASY‐related disorders. Rather than the context‐dependent or compartment‐specific CoA deficits seen in those conditions, PPCS deficiency produces a reproducible reduction of intracellular CoA across patient‐derived models, making total CoA a robust and directly measurable biochemical readout of disease [73]. This distinction has direct therapeutic implications, as it identifies a quantifiable metabolic target and a clear rationale for precursor supplementation.

The cardiac predominance of PPCS deficiency is likely explained by the high energetic demand of the developing and postnatal heart. Patient‐derived iPSC‐cardiomyocytes exhibit impaired contractility, sarcomere disorganization, reduced calcium transients, and arrhythmias, while 3D heart patches show weakened force generation, abnormal force–frequency relationships, and shortened refractory periods [73]. Importantly, the biochemical defect worsens during cardiac maturation, when cardiomyocytes progressively increase reliance on oxidative metabolism and FAO. This provides a possible mechanistic link between PPCS deficiency, tissue‐specific CoA demand, and the selective vulnerability of the developing heart [73]. A muscle biopsy in one case revealed degenerate mitochondria with altered cristae and matrix morphology, together with mild complex IV deficiency, further supporting mitochondrial involvement [74].

Acylcarnitine abnormalities that may mimic very long‐chain acyl‐CoA dehydrogenase deficiency (VLCAD), suggestive of impaired FAO, have been reported during metabolic crises in one severely affected PPCS patient [73]. Unlike the emerging CPT1‐like signature described in COASY‐ and PANK2‐related disease, the PPCS acylcarnitine pattern is not yet sufficiently consistent to be considered a reliable biomarker. However, it is biologically coherent with reduced CoA availability and impaired fatty acid activation, supporting systematic evaluation of dried blood spot and plasma acylcarnitines in future PPCS cases.

The variable expressivity of PPCS deficiency may partly reflect the degree of residual PPCS activity, the cardiac metabolic state, and possibly the gut microbiome or the dietary CoA precursor differences [73]. These findings support a model where PPCS deficiency causes a genuine CoA biosynthetic defect that may be partially tolerated under basal conditions but becomes pathogenic in tissues with high and developmentally increasing demand for CoA‐dependent oxidative metabolism, particularly the heart.

### 3.3 Therapeutic Approaches

Pantethine, a stable disulfide of pantetheine that bypasses the PPCS step, represents the most promising therapy (Figure 2). In four PPCS patients, pantethine administration was associated with sustained clinical stabilization, and patient‐derived models showed increased CoA levels together with partial rescue of sarcomere organization, calcium handling, arrhythmogenicity, and contractility [73]. In a severe infantile case with end‐stage heart failure, treatment at 28 mg/kg/day in four doses dramatically rescued cardiac function, with LVEF normalization within 1 week and sustained stability at 2 years [74]. The therapeutic rationale is therefore stronger than in most other CoA biosynthesis disorders, because the enzymatic block can be bypassed and intracellular CoA depletion is directly measurable. However, available clinical evidence remains limited to very small numbers of patients, and the extent of reversibility likely depends on treatment timing and the degree of established cardiac remodeling. The available data suggest that early initiation is likely critical: pantethine may rescue functional and metabolic defects before irreversible myocardial damage is established, whereas advanced structural remodeling may be only partially reversible. Frequent dosing may also be important, given the need to maintain downstream precursor availability in a metabolically high‐flux tissue [73, 74].

4′‐Phosphopantetheine also raises CoA levels in patient cells, with potential serum stability advantages over pantethine, but its effect appears slightly less efficient in available cellular assays and it has not yet been clinically tested [73]. Thus, while 4′‐phosphopantetheine remains a rational bypass candidate, pantethine currently has stronger disease‐specific clinical support.

These advances position PPCS deficiency among the most therapeutically treatable disorders of CoA biosynthesis, as it combines a measurable CoA deficit, an enzymatic step that can be effectively bypassed, and a main target organ that can achieve rapid and sustained metabolic recovery.

## 4 PPCDC

### 4.1 Clinical Aspects

Phosphopantothenoylcysteine decarboxylase deficiency (PPCDC deficiency) is an ultra‐rare autosomal recessive disorder of CoA biosynthesis, with only one family, comprising two affected sisters, reported to date with confirmed *PPCDC* variants. The disorder presents with severe early‐onset dilated cardiomyopathy, typically beginning in the first days of life and progressing rapidly to a fatal outcome. Additional manifestations include lactic acidosis, elevated creatine kinase, hyperammonemia, liver involvement, lethargy, and limb hypertonia, together with biochemical evidence of systemic metabolic stress [9].

No brain iron accumulation or primary neurodegeneration has been reported, and the overall phenotype closely parallels PPCS deficiency, emphasizing the essential role of 4′‐phosphopantetheine synthesis in cardiac energy metabolism. At present, the clinical spectrum remains extremely narrow, and no controlled natural history data are available.

### 4.2 Metabolic Aspects

PPCDC catalyzes the third step of de novo CoA biosynthesis, namely the FMN‐dependent oxidative decarboxylation of 4′‐phosphopantothenoylcysteine to 4′‐phosphopantetheine. The enzyme is cytosolic and functions as a homotrimer, placing it at critical intermediate step between upstream CoA precursor synthesis and downstream CoA production. In patient‐derived fibroblasts, PPCDC protein is absent, intracellular CoA is reduced to approximately half of control levels, and mitochondrial respiration is impaired, indicating a direct defect in cellular energy metabolism. Unlike the more ambiguous CoA measurements reported in PKAN and some COASY models, PPCDC deficiency, like PPCS deficiency, provides a clearer example in which disruption of an intermediate step of CoA biosynthesis is associated with measurable intracellular CoA depletion, at least in patient fibroblasts.

Functional studies in yeast confirmed the pathogenicity of the reported variants, supporting a causal relationship between *PPCDC* loss and disease. Together with PPCS deficiency, these findings define a cardiometabolic subgroup of CoA biosynthesis disorders in which disruption of the middle steps of the pathway leads to reduced CoA availability and severe cardiac vulnerability.

The biochemical profile of PPCDC patients is notable for hypoglycemia, ketonuria, alanine elevation, urinary dicarboxylic acids, and increased long‐chain acylcarnitines, a pattern suggestive of secondary impairment of FAO or mitochondrial dysfunction. The metabolic abnormalities, mainly acylcarnitine and organic acid changes, are consistent with secondary fatty‐acid oxidation impairment, likely reflecting insufficient CoA availability for fatty‐acid activation. At present, no mammalian animal model has been described, which limits mechanistic dissection and preclinical therapeutic testing.

### 4.3 Therapeutic Approaches

Therapeutic experience in PPCDC deficiency is very limited, but pantethine is the most rational pathway‐targeted option because it bypasses both the PPCS and PPCDC enzymatic steps (Figure 2). One patient with PPCDC deficiency was treated with pantethine and showed encouraging but still limited improvement, providing preliminary clinical support for this strategy. Additional support for this approach comes from in vitro and bacterial studies, where pantethine rescues coaBC‐deleted *E. coli* , a model lacking both PPCS and PPCDC activities.

The favorable rescue observed in PPCS deficiency further supports early pantethine supplementation as a rational approach in PPCDC deficiency. However, the available clinical evidence remains anecdotal, and no controlled studies or approved therapies exist. 4′‐phosphopantetheine is also mechanistically attractive because it bypasses PPCDC directly and enters CoA biosynthesis downstream of the enzymatic block [76].

For now, PPCDC deficiency should be considered a severe but poorly characterized and potentially pathway‐responsive CoA biosynthesis disorder in which early metabolic intervention may be the best available therapeutic direction.

## 5 COASY

### 5.1 Clinical Aspects

Biallelic pathogenic variants in *COASY* cause a spectrum of COASY‐related disorders that includes COASY protein‐associated neurodegeneration (CoPAN; OMIM #615643), pontocerebellar hypoplasia type 12 (PCH12; OMIM #618266), and riboflavin‐responsive lipid storage myopathy (RR‐LSM). CoPAN was initially described as an ultra‐rare NBIA disorder characterized by clinical signs similar to those of PKAN, namely childhood‐onset progressive dystonia, oromandibular spasticity, parkinsonism, gait impairment, cognitive decline, and neuropsychiatric manifestations, often associated with iron deposition in the basal ganglia [13, 77, 78]. Subsequent reports have considerably expanded this picture. Recently described patients showed atypical manifestations, such as epilepsy, hearing loss, speech and language delay, autism spectrum disorder, pigmentary retinopathy, neuromuscular involvement, microcephaly, brain atrophy, and brainstem or basal ganglia changes, often without detectable iron depositions [15, 79]. Unlike PKAN, basal ganglia iron accumulation is therefore not a consistent neuroradiological feature of COASY‐associated disease, where developmental brain abnormalities and atrophy may prevail.

PCH12 represents the severe end of the clinical spectrum associated with COASY deficiency and is generally linked to biallelic *COASY* variants causing complete loss of COASY function. It presents with microcephaly, pontocerebellar hypoplasia, arthrogryposis, severe neurodevelopmental impairment, and perinatal or early infantile lethality, without brain iron accumulation [80, 81, 82]. More recently, biallelic *COASY* variants were identified as a cause of previously unsolved RR‐LSM, a muscle‐predominant disorder characterized by exercise intolerance or weakness, lipid droplet accumulation in myofibers, occasional multisystem involvement, and responsiveness to riboflavin [10]. Together, these observations suggest a continuum model in which COASY‐related phenotypes may reflect the combined effects of residual protein function, developmental timing, and tissue‐specific metabolic demand, rather than discrete disease entities.

### 5.2 Metabolic Aspects

COASY is the bifunctional enzyme catalyzing the final two reactions of the de novo CoA biosynthesis, through phosphopantetheine adenylyltransferase (PPAT) and dephospho‐CoA kinase (DPCK) activities [83]. Although COASY has historically been described as mainly mitochondrial [13, 84, 85], with additional cytosolic and nuclear localization reported [86], recent evidence suggests that the majority of endogenous COASY is cytosolic in cultured human cells, with only a minor fraction associated with mitochondria [87]. In the same study, de novo CoA biosynthesis was found to occur predominantly in the cytosol, where it supports lipid anabolism, whereas the mitochondrial CoA pool largely depended on SLC25A16‐ and SLC25A42‐mediated import of free CoA to sustain mitochondrial catabolic pathways, including the TCA cycle and FAO [87]. These findings challenge a simple localization‐based explanation for the neurological vulnerability of COASY disorders and suggest that disease may arise from impaired compartment‐specific CoA handling and insufficient flux through selected CoA‐dependent processes, rather than from mitochondrial COASY deficiency alone.

As in PKAN, total CoA measurements do not fully explain pathogenesis. Patient fibroblasts and neuronal Coasy‐deficient mouse brains showed near‐normal total CoA levels despite clear mitochondrial and cellular abnormalities [13, 14, 15, 88]. In contrast, CoA reduction has been reported in other experimental systems, including COASY‐depleted human cancer cell line and zebrafish embryos [86, 87, 89]. Interestingly, fibroblasts of patients with pathogenic *COASY* variants showed reduced amounts of mitochondrial 4′‐phosphopantetheinylated proteins, including mtACP and ALDH1L2, despite the apparent unchanged levels of total CoA [15]. This links COASY dysfunction to impaired 4′‐phosphopantetheinylation, mitochondrial respiratory defects, and altered mitochondrial integrity, providing a mechanistic bridge with PKAN.

Recent newborn screening studies further support a systemic metabolic component. Patients with CoPAN or PCH12 may show a dried blood spot acylcarnitine profile resembling CPT1 deficiency, with elevated free carnitine and increased C0/(C16 + C18) ratios, often followed by normal or only mildly abnormal plasma acylcarnitine profiles [79, 90, 91]. This signature remains mechanistically unresolved, but it may reflect impaired CoA‐dependent fatty acid activation or altered acyl‐CoA/acylcarnitine balance. If prospectively validated, it could provide an early diagnostic clue and support inclusion of COASY‐related disorders in the differential diagnosis of newborn screening patterns suggestive of CPT1 deficiency.

The pathogenic consequences of COASY deficiency are consistent across disease‐relevant models, converging on mitochondrial dysfunction, iron dyshomeostasis, and lipid alteration. Patient fibroblasts showed impaired mitochondrial oxygen consumption and transcriptomic dysregulation involving, among others, oxidative phosphorylation, oxidative stress responses, and lipid‐related pathways [15]. Astrocytes differentiated from patient‐derived iPSC displayed iron overload, increased labile iron, impaired tubulin acetylation, mitochondrial abnormalities, defective vesicular trafficking, lipid peroxidation, and ferroptosis [92]. In vivo, the severity of the phenotype aligns with the degree of COASY loss and with its developmental timing. Constitutive *Coasy* knock‐out is embryo lethal in mouse, mirroring the severity of human PCH12, while the congenic conditional neuronal ablation induced a very severe early‐onset phenotype characterized by motor impairment, growth arrest, dystonia‐like features, mitochondrial abnormalities, and premature death [14]. However, inducible neuronal deletion of *Coasy* resulted in a more progressive CoPAN‐like phenotype, characterized by motor decline, neurodegeneration, brain iron dyshomeostasis, astrogliosis and microglial activation, thereby identifying neuroinflammation as a potential additional driver of disease progression [88]. The developmental role of *COASY* is highlighted by the glial deletion mouse model, in which conditional *Coasy* loss leads to cortical and cerebellar hypoplasia, abnormal neuronal development, astrocytic hyperproliferation, lipid peroxidation, iron dyshomeostasis, and impaired mitochondrial respiration [93]. This model provides a mechanistic explanation for the neurodevelopmental features of PCH12 and of atypical CoPAN, positioning *COASY* at the intersection of neurodevelopment and neurodegeneration.

### 5.3 Therapeutic Approaches

No disease‐modifying treatment is currently available for CoPAN or PCH12 (Figure 2). In contrast to PANK2, PPCS, and PPCDC deficiencies, precursor‐bypass strategies are unlikely to provide meaningful benefit because the enzymatic block lies at the final step of CoA biosynthesis [17, 94]. Indeed, pantethine or 4′‐phosphopantetheine cannot bypass COASY deficiency, since their conversion to CoA remains dependent on functional PPAT and DPCK activities [24]. Exogenous CoA produced beneficial effects in vitro on PKAN [42, 43, 48, 49] and CoPAN [92] cellular models, and was also able to restore normal development in a zebrafish *coasy* model [89], suggesting that CoA‐derived metabolites may enter salvage routes. However, because extracellular CoA is poorly membrane‐permeable and rapidly degraded to 4′‐phosphopantetheine, its therapeutic relevance in COASY deficiency remains uncertain and likely depends on residual COASY activity [59, 95].

Therefore, therapeutic strategies for COASY‐related disorders may need to enhance or genetically correct COASY (dys)function, stabilize specific mutant proteins, or target downstream pathological processes rather than simply provide upstream precursors. In the inducible neuronal mouse model, the brain‐penetrant PPARγ agonist leriglitazone improved motor performance, restored iron homeostasis, reduced neuroinflammation, and attenuated neurodegeneration, without rescuing survival or correcting the primary biosynthetic defect [88]. Leriglitazone therefore targets neuroinflammation, mitochondrial dysfunction, lipid metabolism, and iron handling as convergent downstream consequences of impaired CoA‐dependent metabolism, rather than addressing the upstream defect itself. Consistent with findings in PKAN patient‐derived astrocytes, these results support PPARγ activation as a promising downstream therapeutic strategy for these conditions and potentially other NBIA disorders that share neuroinflammation, mitochondrial dysfunction, and iron dyshomeostasis.

Riboflavin represents a distinct, variant‐specific therapeutic strategy. In COASY‐related RR‐LSM, the recurrent K371R variant affects the DPCK domain, reduces COASY protein abundance and activity, and causes muscle lipid droplet accumulation under lipid overload. Riboflavin, as a FAD precursor, appears to stabilize the mutant protein, restore CoA levels in knock‐in flies, reduce lipid accumulation, improve locomotor performance, and produce clinical benefit in affected individuals [10]. However, this mechanism is specific to hypomorphic alleles that retain residual, chaperone‐responsive function, and it cannot be generalized to null variants or to other COASY‐related phenotypes.

## 6 Common Points, Divergences, and Perspectives

### 6.1 Common Metabolic Themes

The four inherited disorders of CoA biosynthesis clearly show how defects in this single linear pathway can generate markedly divergent diseases, indicating that pathway position contributes to, but does not determine, disease expression. More importantly, the shared CoA biosynthesis pathway does not necessarily translate into a shared biochemical defect, particularly if the latter is defined only as reduced total cellular CoA. While PPCS and PPCDC deficiencies appear more directly associated with decreased CoA availability, total CoA levels are often preserved in PKAN and COASY‐related disorders under basal conditions. This discrepancy suggests that steady‐state CoA abundance is an incomplete readout of the pathway function, while the pathogenic defect may emerge when cells are unable to sustain CoA‐dependent flux under conditions of increased metabolic demand.

This view is reinforced by the compartmentalized nature of CoA metabolism. CoA and acyl‐CoA species are distributed across mitochondria, cytosol, peroxisomes, and nucleus, where they support distinct reactions, including FAO, the TCA cycle, lipid biosynthesis, protein acylation and 4′‐phosphopantetheinylation. Thus, preserved total CoA may mask functionally relevant defects in selected compartments, cell types, or metabolic states.

A related theme is the perturbation of lipid metabolism and acyl‐group homeostasis. CPT1‐like acylcarnitine profiles in COASY‐related disorders and PKAN suggest that altered CoA availability may affect long‐chain fatty acid activation or acyl‐CoA/acylcarnitine balance. Although these profiles are not disease‐specific and their mechanistic basis remains unclear, they indicate a systemic metabolic component that extends beyond the clinically predominant tissue. Similarly, data suggesting a specific requirement for PANK2 during the metabolic switch toward FAO support the idea that at least PKAN‐ and CoPAN‐associated disorders impair metabolic adaptability rather than just basal CoA production.

Impaired 4′‐phosphopantetheinylation of selected metabolic proteins may represent another point of convergence. Reduced levels of 4′‐phosphopantetheinylated mitochondrial proteins in *PANK2*‐ and *COASY*‐mutant fibroblasts, despite preserved total CoA, provide a potential mechanistic link between defective CoA biosynthesis, mitochondrial dysfunction, and lipid‐related metabolism. Whether similar defects contribute to PPCS and PPCDC deficiencies remains an important open question.

### 6.2 Explaining Disease Divergence

A major unresolved question is why defects within the same biosynthetic pathway produce predominantly neurological disease in *PANK2* and *COASY* variants, but severe cardiomyopathy in *PPCS* and *PPCDC* deficiencies. Current evidence suggests that disease phenotype reflects a complex interplay among residual enzymatic activity, developmental timing, tissue‐specific metabolic demand, and compartment‐specific CoA requirements.

In this context, an additional and often underappreciated dimension is the distinct functional role of individual enzymes within the pathway. PANK2 and COASY, which catalyze the first and final steps of CoA biosynthesis, respectively, have also been implicated in pathway regulation and metabolic sensing, whereas PPCS and PPCDC catalyze intermediate steps for which no clear regulatory function has yet been established.

This functional asymmetry suggests that enzyme position may influence disease expression not only through the site of the biochemical block, but also through the capacity of each step to regulate pathway flux. As the rate‐limiting enzyme at the entry point of the pathway, PANK2 is strategically positioned to integrate metabolic cues and regulate CoA flux, particularly within mitochondria.

In PKAN, the selective vulnerability of the GP remains incompletely understood, but may reflect the dependence of specific neuronal populations on tightly controlled acyl‐CoA metabolism and mitochondrial lipid handling. PANK2 is the only PANK isoform associated with mitochondria and appears particularly relevant under lipid‐dependent metabolic conditions. This may make specific neuronal populations vulnerable when mitochondrial fatty acid handling, membrane remodeling, or acyl‐CoA‐dependent reactions become limiting. The presence of compensatory PANK isoforms and the lack of consistent basal CoA depletion argue against a simple model of constitutive deficiency and instead support the hypothesis that dysregulation of CoA flux may play a key role in neuronal susceptibility.

At the distal end of the pathway, COASY catalyzes the final step of CoA synthesis and may represent another critical point at which biosynthetic capacity, compartmental CoA supply, and metabolic utilization converge. COASY‐related disorders further demonstrate how residual enzymatic activity and developmental timing shape phenotypic outcome. Complete loss of COASY function leads to the severe neurodevelopmental phenotype of PCH12, whereas partial loss of function results in CoPAN, supporting a phenotypic continuum where nervous system vulnerability may reflect not only high energy requirements but also the dependence of neurodevelopment, axonal maintenance, membrane metabolism, and glial‐neuronal interactions on CoA‐dependent processes.

PPCS and PPCDC deficiencies affect intermediate catalytic steps without a currently established regulatory role and are predominantly associated with cardiomyopathy. The postnatal heart undergoes a progressive metabolic shift toward oxidative metabolism and FAO, increasing its dependence on sustained CoA availability. Under these conditions, even partial impairment of CoA biosynthesis may become deleterious during cardiomyocyte maturation, when CoA‐dependent oxidative flux is critical for contractile function. Consistently, patient‐derived PPCS cardiac models support this view, showing that biochemical and functional defects become more pronounced with maturation, supporting the notion that the heart represents a high‐flux system with limited buffering capacity for CoA deficiency.

Overall, these observations support a model in which the position of each enzyme within the pathway, together with its regulatory properties, contributes to a continuum of tissue vulnerability: upstream regulatory defects (PANK2) predominantly affect the nervous system, intermediate catalytic defects (PPCS and PPCDC) preferentially impact the heart, and downstream integrative defects (COASY) give rise to broader, multisystem phenotypes involving both the brain and peripheral tissues such as skeletal muscle. This framework provides a unifying perspective to interpret phenotypic heterogeneity across CoA biosynthesis disorders and highlights the importance of considering both enzymatic function and network‐level regulation in disease pathogenesis.

### 6.3 Diagnostic and Therapeutic Perspectives

The diagnostic approach to inherited disorders of CoA biosynthesis remains largely gene‐driven, but emerging metabolic signatures may support earlier recognition or functional stratification. Acylcarnitine abnormalities are particularly relevant. Newborn screening profiles resembling CPT1 deficiency have been reported in COASY‐related disorders, and similar patterns have now been described also in PKAN. Although prospective validation is needed, these findings suggest that *PANK2* and *COASY* should be considered in unexplained CPT1‐like dried blood spot profiles, especially when *CPT1A* testing is negative or clinical features are atypical. Whether comparable signatures occur in PPCS and PPCDC deficiencies remains unclear. Current evidence suggests that such profiles are not consistently present in PPCS deficiency.

Future diagnostic work should move beyond steady‐state total CoA measurements. Acyl‐CoA profiling, compartment‐resolved metabolomics, assessment of 4′‐phosphopantetheinylated proteins, and functional assays under metabolic stress may reveal defects that are silent under basal conditions, thereby supporting both variant interpretation and pharmacodynamic monitoring in therapeutic studies.

Therapeutically, these disorders require pathway‐specific strategies. In PANK2, PPCS, and PPCDC deficiencies, bypass or boosting approaches remain conceptually attractive because downstream precursors such as pantethine or 4′‐phosphopantetheine can, in principle, enter the pathway beyond selected enzymatic blocks. The strongest current rationale is in PPCS deficiency, where pantethine has been associated with clinical improvement or stabilization in a small number of patients. However, reversibility is likely constrained by established cardiac remodeling, supporting early diagnosis and treatment.

For PKAN, PANK activation and precursor‐based strategies aim to restore pathway flux or compensate for PANK2 dysfunction, although clinical efficacy remains uncertain. In COASY‐related disorders, precursor bypass is less straightforward because COASY catalyzes the final steps of CoA formation. Pantethine or 4′‐phosphopantetheine would still require residual COASY activity, making downstream metabolic modulation, variant‐specific stabilization, or gene‐based strategies more rational.

In conclusion, while reliable methods to measure intracellular and compartment‐specific CoA pools remain an unmet need, the field should move from asking whether total CoA is reduced to identifying which CoA‐dependent reactions fail, in which compartment, and under which metabolic conditions. This view may help explain why defects in the same pathway lead to different diseases and may guide the development of biomarkers and therapies tailored to the specific metabolic defect in each disorder.

## Author Contributions

I.D.M. and A.I. conceptualized the review and prepared the original draft. Y.A. revised the clinical descriptions. V.T. contributed to the development of the discussion section. All authors reviewed, revised, and approved the final version of the manuscript for submission.

## Funding

This study was supported by the Bavarian University Support Program for the Establishment of International Research Collaborations (BayIntAn_TUM_MRI_2025_03) to A.I. and by the Ricerca Corrente (RRC) to the Fondazione IRCCS Istituto Neurologico Carlo Besta.

## Conflicts of Interest

The authors declare no conflicts of interest.

## Data Availability

Data sharing not applicable to this article as no datasets were generated or analyzed during the current study.
